# The impact of surgery with general anesthesia on cognitive function and putamen volume: a cross-sectional study among older adults

**DOI:** 10.3389/fnagi.2024.1483989

**Published:** 2024-12-09

**Authors:** Jianjun Jiang, Zhuyun Zhang, Hong Zheng, Jian Lu, Wei Li

**Affiliations:** ^1^Department of Anorectal, KongJiang Hospital of Yangpu District, Shanghai, China; ^2^Department of General Surgery, Yangpu Hospital, School of Medicine, Tongji University, Shanghai, China; ^3^Shanghai Changing Mental Health Center, Affiliated Mental Health Center of East China Normal University, Shanghai, China; ^4^Department of Geriatric Psychiatry, Shanghai Mental Health Center, Shanghai Jiao Tong University School of Medicine, Shanghai, China

**Keywords:** surgery, general anesthesia, MRI, putamen, 2 cognitive function, older adults

## Abstract

**Background:**

Previous studies have shown that surgery under general anesthesia may diminish cognitive function; however, the proposed mechanisms need further elucidation. The purpose of the current study was twofold: (1) to compare overall and domain-specific differences in cognitive function between the surgery under general anesthesia group and the control group, and (2) to investigate the possible mechanisms of surgery under general anesthesia affecting cognitive function, using T1-structural magnetic resonance imaging.

**Methods:**

A total of 194 older adults were included in this study. Patients were divided into a surgery under general anesthesia group (*n* = 92) and a control group (*n* = 104). The two groups were matched for age, sex, and educational level. All participants underwent clinical evaluation, neuropsychological testing, blood biochemistry analysis, and T1 phase structural magnetic resonance imaging.

**Results:**

We found that older adults with a history of surgery under general anesthesia had lower Montreal Cognitive Assessment (MoCA) scores and smaller right putamen volumes (*p* < 0.05). Linear regression analysis (mediation model) indicated that surgery under general anesthesia affected MoCA scores by diminishing the volume of the right putamen (B = 1.360, *p* = 0.030).

**Conclusion:**

We found evidence that older adults who underwent surgery under general anesthesia had poorer cognitive function, which may have been caused by an apoptotic or otherwise toxic effect of anesthetic drugs on the volume of the right putamen.

## 1 Introduction

Alzheimer’s disease (AD), the leading cause of dementia, has rapidly become one of the most expensive, deadly, and burdensome diseases of this century (Scheltens et al., 2021). The core pathological features of Alzheimer’s disease are amyloid plaques and neurofibrillary tangles (NFTs), and factors such as immunity, inflammation, and infection may also contribute to the pathogenesis of AD (Serrano-Pozo et al., 2011). Currently, available treatments for AD include cholinesterase inhibitors and memantine. These drugs have been shown to improve the patients’ quality of life; however, they do not change the course of the disease or its rate of decline (Mossello and Ballini, 2012). Therefore, we are more concerned with early prevention than late treatment.

As the age of an individual increases, the probability of undergoing surgery under general anesthesia also increases. A substantial body of evidence from both *in vivo* and *in vitro* models suggests that exposure to anesthetics may increase the risk of AD through a mechanism similar to its neuropathology (Tsolaki et al., 2024). In addition, epidemiological studies have shown that surgery under general anesthesia is likely to increase the risk of AD. For instance, in a study by Vanderweyde et al. (2010), prostate or hernia surgery increased the risk of dementia, especially AD (Vanderweyde et al., 2010). In a nine-year follow-up of 3,100 patients, Sohn et al. found that the overall incidence of dementia was higher in those who underwent surgery under general anesthesia than in the control group (10.5 versus 8.8 per 1,000 person-years), especially among women or individuals with combined medical conditions (Sohn et al., 2021). However, a recent meta-analysis of case–control studies (*N* = 15) found no association between AD and prior exposure to surgery under general anesthesia (OR = 1.05, 95% CI: 0.93–1.19, *p* = 0.4) (Seitz et al., 2013; Seitz et al., 2011). Therefore, the link between surgery under general anesthesia and dementia needs to be further studied.

Magnetic resonance imaging (MRI) is an effective tool for studying brain and cognitive function and helps reveal the possible mechanisms through which anesthesia affects cognition. In animal studies, researchers have found that the putamen is unusually sensitive to narcotic drugs, such as sevoflurane (Burks et al., 2020). In another animal experiment, the researchers found that Fluoro-Jade C staining in the caudate putamen of mice was significantly elevated after sevoflurane exposure (Walters et al., 2020). Moreover, one study showed that when normal individuals undergo surgery under general anesthesia, blood flow in the putamen is significantly reduced (Schlünzen et al., 2007). Therefore, we speculate that the putamen is likely the target of cognitive decline induced by surgery under general anesthesia.

To test the above research hypothesis, we recruited 92 community-dwelling older adults who had undergone surgery under general anesthesia and 102 normal controls who were matched for age, sex, and education. All participants completed blood biochemical, neuropsychological, and T1 structural magnetic resonance tests. We hypothesized that: (1) older adults who have undergone surgery under general anesthesia may have poorer cognitive function, and (2) structural changes in the putamen may play an important regulatory role in the process of anesthesia-induced cognitive decline.

## 2 Materials and methods (Li et al., 2022)

### 2.1 Participants

This cross-sectional study was conducted at the KongJiang Hospital of Yangpu District between March 1, 2023 and April 1, 2024. The inclusion criteria were as follows: (1) aged 55 years and above; (2) without significant cognitive impairment before surgery, such as mild cognitive impairment (MCI) or dementia; (3) without obvious visual or hearing impairment; (4) T1-structural magnetic resonance tests. The exclusion criteria were as follows: (1) aged below 55 years; (2) non-surgery with general anesthesia, such as surgery with local anesthesia; (3) chronic diseases that may affect cognitive function, such as dementia, major depressive disorder, and schizophrenia; and (4) complicated by serious physical diseases, such as myocardial infarction, cerebral infarction, and cerebral hemorrhage. Simultaneously, we recruited a group of normal controls from the Yangpu community who had not undergone surgery under general anesthesia; their inclusion and exclusion criteria were the same as before. To exclude the effects of age, sex, and education on cognitive function, we matched the variables between the two groups.

All participants signed an informed consent form before the study was initiated, and ethical approval was obtained from the Ethics Committee of KongJiang Hospital of Yang Pu District.

### 2.2 Sample size evaluation

Under previous magnetic resonance imaging (fMRI) protocols, a sample size of 30 cases per group has become the “minimum requirement” and common choice for confirmatory studies (Zhou et al., 2024). Since we also needed to match sex, age, and education level, we needed at least 87 participants per group after calculating through the Power Analysis and Sample Size (PASS) software. Finally, we enrolled 92 surgical patients and 102 normal controls, matched for sex (males: 39 (42.4%) vs. 57 (55.9%), *p* = 0.064), age (70.02 ± 7.206 vs. 68.52 ± 7.470, *p* = 0.157), and years of education (8.44 ± 4.452 vs. 9.51 ± 3.601, *p* = 0.071). The results are summarized in Table 1.

**Table 1:** Comparison of general demographic data, blood biochemical markers, neuropsychological tests, and brain structure between surgery and non-surgery individuals.

Variables	Surgery (*n* = 92)	Non-surgery (*n* = 102)	X^2^ or t	*p*	cohen’d
Age, y	70.02 ± 7.206	68.52 ± 7.470	1.422	0.157	0.010
Education, y	8.44 ± 4.452	9.51 ± 3.601	−1.814	0.071	0.017
Male, *n*(%)	39(42.4)	57(55.9)	3.522	0.064	0.018
Smoker, *n*(%)	26(28.3)	32(31.4)	0.223	0.754	0.001
Drinker, *n*(%)	16(17.4)	22(21.6)	0.536	0.476	0.003
Hypertension, *n*(%)	44(47.8)	51(50.0)	0.091	0.776	<0.001
Diabetes, *n*(%)	17(18.5)	19(18.6)	0.001	1.000	<0.001
Blood biochemical markers					
Fasting blood glucose, mmol/L	1.35 ± 0.71	1.52 ± 0.75	−1.231	0.221	0.002
Triglycerides, mmol/L	1.51 ± 0.87	1.44 ± 0.79	0.434	0.665	0.004
High-density lipoprotein, mmol/L	1.46 ± 0.82	1.26 ± 0.67	1.340	0.183	0.010
Low-density lipoprotein, mmol/L	1.66 ± 0.85	1.51 ± 0.70	0.932	0.354	<0.001
Apoprotein A	1.80 ± 0.86	1.64 ± 0.77	1.034	0.303	0.007
Neuropsychological tests					
MoCA	23.34 ± 4.983	24.94 ± 3.612	−2.528	0.012*	0.034
Digit span	14.60 ± 4.299	15.11 ± 3.857	−0.888	0.376	0.004
Auditory word learning test	32.23 ± 8.982	32.17 ± 9.320	0.045	0.964	<0.001
Associative learning test	6.55 ± 3.407	6.58 ± 3.169	−0.072	0.943	<0.001
Visual recognition function test	3.41 ± 0.869	3.62 ± 0.630	−1.912	0.058	0.019
Language fluency	26.98 ± 8.226	28.58 ± 9.771	−1.225	0.222	0.008
Webster’s mapping	10.47 ± 3.53	10.83 ± 4.483	−0.627	0.532	0.002
Wechsler block diagram	27.26 ± 8.097	27.89 ± 8.034	−0.554	0.587	0.002
Brain structure					
Total brain volume, cm^3^	1443.68 ± 149.390	1464.90 ± 145.76	−0.996	0.321	0.005
Left hippocampus, cm^3^	3.566 ± 0.430	3.660 ± 0.431	−1.510	0.133	0.012
Right hippocampus, cm^3^	3.742 ± 0.501	3.877 ± 0.482	−1.902	0.059	0.018
Left putamen, cm^3^	4.425 ± 0.564	4.578 ± 0.662	−1.717	0.088	0.015
Right putamen, cm^3^	4.444 ± 0.571	4.660 ± 0.658	−2.439	0.016*	0.030

*means *p* < 0.05; MoCA means montreal cognitive assessment.

### 2.3 Clinical assessment and general demographic data collection

All participants completed a series of clinical assessments, physical examinations, and general demographic surveys. Through face-to-face interviews, we obtained general demographic data (age, sex, and education), daily living habits (smoking and drinking), and disease history (hypertension and diabetes). At the same time, we also investigated information about surgery with general anesthesia, including the age at surgery, the type of surgery as well as the main drugs used for surgery under general anesthesia.

### 2.4 Neuropsychological assessment

All participants completed a series of neuropsychological tests, including the Montreal Cognitive Assessment (MoCA) (Nasreddine et al., 2005), digit span (Leung et al., 2011), auditory word learning test (Hong et al., 2012), associative learning test, visual recognition function test, verbal fluency tasks (Aita et al., 2019), Webster’s mapping, and Wechsler block diagram (Li et al., 2017). The cognitive areas assessed in these scales are: overall cognitive function, attention and short-term memory, auditory memory, association and reaction speed, visual ability to distinguish numbers, letters and words, language ability, semantic memory and executive function. All scales were completed by professionally trained psychological testers and consistency training was conducted to ensure accuracy and consistency of the scale assessment.

### 2.5 Biochemical indexes

After an overnight fast, peripheral blood samples were collected between 7 a.m. and 9 a.m. Serum separation tubes containing activated clot gel were used to detect biochemical indices. Fasting blood glucose, triglyceride, high-density lipoprotein, low-density lipoprotein, and apoprotein A levels were measured using an Olympus AU2700 automatic biochemical analyzer (Beckman Coulter, Inc., Carlsbad, CA, United States).

### 2.6 T1 phase structure magnetic resonance imaging

Structural images of the brain were captured using a Magnetom Verio 3.0 T scanner (Siemens, Munich, Germany). The sequence parameters of the rapid gradient echo (MPRAGE) prepared using T1-weighted three-dimensional magnetization were as follows: TR = 2,300 ms, TE = 2.98 ms, matrix size = 240 × 256, turning angle = 9 °, film thickness = 1.2 mm, and field of view (FOV) = 240 × 256 mm. As described by Wolz et al., volume data was evaluated by an automated procedure (Wolz et al., 2014). Using FreeSurfer, we obtained the participants’ whole-brain, hippocampal, and caudate putamen volumes. In addition, to evaluate the impact of left–right differences, the asymmetry index was calculated as [right-to-left volume]/[total volume] × 100%. Quality control was ensured by overlapping the output packages on FreeSurfer templates and visual evaluations were performed.

## 3 Statistical analysis

Continuous variables are expressed as mean ± standard deviation (SD), and categorical variables are expressed as frequency (%). The one-sample Kolmogorov–Smirnov test was used to test whether the data conformed to a normal distribution. Independent sample t-tests and Kruskal-Wallis H tests were used to compare normal and non-normal data between the surgical and non-surgical groups, respectively. Chi-square tests were used to compare the classification variables. Next, correlation and linear regression analyses (mediating model) were used to investigate the associations between surgery and general anesthesia, cognitive-related brain areas, and cognitive scores. A two-tailed test was used for all analyses, and the significance level was set at *p* < 0.05. SPSS 22.0 (IBM Corporation, Armonk, NY, United States) was used for data analysis.

## 4 Results

### 4.1 Characteristics of subjects with different surgical conditions

Participants who underwent surgery under general anesthesia had lower overall MoCA scores and a smaller volume of the right putamen than participants who did not undergo surgery under general anesthesia (*p* < 0.05), while there was no statistical difference (*p* > 0.05) related to age, education, sex, smoking and drinking status, hypertension, diabetes, fasting blood glucose, triglyceride, high-density lipoprotein, low-density lipoprotein, apoprotein A, digit span, auditory word learning, associative learning, visual recognition function, language fluency, Webster’s mapping, Wechsler block diagram, total brain volume, left hippocampus, right hippocampus, and left putamen between the two groups. Table 1 shows the results.

### 4.2 The connection between surgery under general anesthesia and brain structure

**Figure 1:**
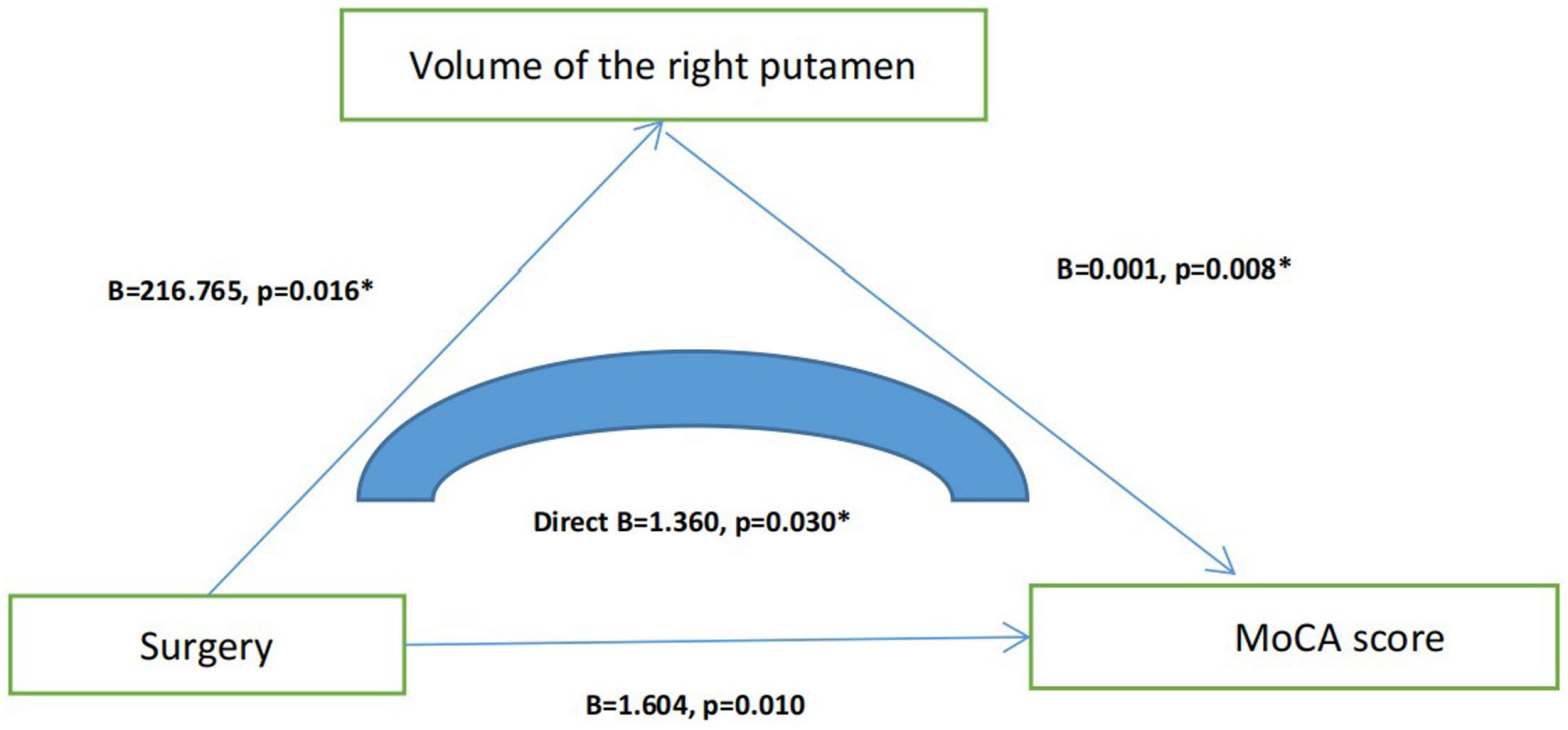
Mediating effect model among surgery, right putamen volume and MoCA score.

To explain the possible mechanisms through which surgery under general anesthesia affects the overall cognitive function, we added T1-structural magnetic resonance data. We found that older adults with a history of surgery under general anesthesia had lower MoCA scores and smaller right putamen volumes (*p* < 0.05). Using correlation analysis, we found that the volume of the right amygdala was significantly correlated (*p* = 0.008, r = 0.189) with MoCA. Using linear regression analysis (mediation model), we found that surgery under general anesthesia directly affected the MoCA score by affecting the volume of the right putamen (B = 1.360, *p* = 0.030) (Figure 1).

## 5 Discussion

The purpose of this study was to investigate the relationship between surgery under general anesthesia and cognitive decline, and to explore the possible mechanism through which surgery under general anesthesia affects cognitive function. In this study, we recruited 92 community-dwelling older adults who underwent surgery under general anesthesia and 102 healthy controls matched for age, sex, and education. We found that: (1) older adults who underwent surgery under general anesthesia had poorer overall cognitive function, and (2) surgery under general anesthesia may affect cognitive function by affecting the volume of the right putamen, and there might be a causal relationship between the three factors (surgery under general anesthesia, right putamen, and cognitive function).

Several studies have explored the relationship between surgery under general anesthesia and cognitive function. Yu et al. found that whether propofol or sevoflurane was used in thoracic surgery, it would damage the cognitive function of older adults (Yu, 2017). Meng et al. found that surgery under general anesthesia and epidural anesthesia combined with general anesthesia can both damage the overall cognitive function of older adults with liver cancer (Meng et al., 2021). Moreover, Zhang et al. found that in addition to overall cognitive function, surgery under general anesthesia can also impair social cognitive function in older adults (Zhang et al., 2020). Therefore, our findings are consistent with these results.

To further explore the possible mechanisms through which surgery under general anesthesia affects overall cognitive function, we included T1 phase magnetic resonance data. We recruited two groups of older adults matched for age, sex, and education and ultimately found that individuals who underwent surgery under general anesthesia had poorer overall cognitive function than those who did not, while at the same time having a smaller right putamen volume. Using linear regression analysis (mediation model), we found that surgery under general anesthesia directly affected the MoCA score by affecting the volume of the right caudate putamen. The caudate putamen is part of the striatum and a component of the external vertebral system. Previous studies have shown that the anatomical connectivity, functional specialization, and neurochemical characteristics of the caudate putamen in patients with AD are significantly different from those in healthy controls (Selden et al., 1994). Moreover, other studies have confirmed a significant reduction in the putamen volume in patients with AD (Yoo et al., 2020; Cogswell et al., 2021). Therefore, we speculated that the putamen may also be involved in the pathogenesis of AD. However, there are few studies on the relationship between surgery under general anesthesia and the putamen. Further investigation is needed to determine whether anesthetic drugs affect cognitive function by affecting the putamen.

## 6 Limitations

Our study has some limitations. First, it is only a cross-sectional study that cannot establish a cause-and-effect relationship between surgery under general anesthesia and cognitive decline. Second, the relatively small sample size reduces the reliability of the study. Third, there are many confounding factors, such as different types of surgery, different choices of anesthetic drugs, and different durations of anesthesia, which may have affected the results. Fourth, our current study focuses only on the relationship between the putamen and surgery under general anesthesia without considering the impact of other cognitive brain regions, such as the hippocampus and amygdala, on the results, which is perhaps the biggest limitation of our study. Fifth, a recent review highlighted that stressful life events can also lead to cognitive deficits and even AD (not anesthesia itself, but the process of going to the hospital) (Martin et al., 2024). In addition, it is difficult to separate the effects of anesthesia from the effects of surgery on cognition, which is a major limitation of the current study (Cottrell and Hartung, 2020). Therefore, we plan to focus on solving these problems in future studies.

## 7 Conclusion

Surgery under general anesthesia may impair overall cognitive function in older adults, and the mechanism may be related to its effect on the right putamen volume.

## Data Availability

The raw data supporting the conclusions of this article will be made available by the authors, without undue reservation.
